# TDP-43 Cytoplasmic Translocation in the Skin Fibroblasts of ALS Patients

**DOI:** 10.3390/cells11020209

**Published:** 2022-01-08

**Authors:** Miguel A. Rubio, Mireia Herrando-Grabulosa, Roser Velasco, Israel Blasco, Monica Povedano, Xavier Navarro

**Affiliations:** 1 Neuromuscular Unit, Department of Neurology, Hospital del Mar, 08003 Barcelona, Spain; marubio@psmar.cat; 2 Department of Cell Biology, Physiology and Immunology, Institute of Neurosciences and CIBERNED, Universitat Autònoma de Barcelona, 08193 Bellaterra, Spain; mireia.herrando@uab.cat (M.H.-G.); francast2@msn.com (R.V.); israel.blasruan@gmail.com (I.B.); 3 Neuro-Oncology Unit, Department of Neurology, Hospital Universitari de Bellvitge-ICO and IDIBELL, 08907 L’Hospitalet, Spain; 4 Department of Neurology, Hospital Universitari de Bellvitge, 08907 L’Hospitalet, Spain; 30058mpp@gmail.com

**Keywords:** amyotrophic lateral sclerosis, dermis, skin biopsy, TDP-43, biomarker

## Abstract

Diagnosis of ALS is based on clinical symptoms when motoneuron degeneration is significant. Therefore, new approaches for early diagnosis are needed. We aimed to assess if alterations in appearance and cellular localization of cutaneous TDP-43 may represent a biomarker for ALS. Skin biopsies from 64 subjects were analyzed: 44 ALS patients, 10 healthy controls (HC) and 10 neurological controls (NC) (Parkinson’s disease and multiple sclerosis). TDP-43 immunoreactivity in epidermis and dermis was analyzed, as well as the percentage of cells with TDP-43 cytoplasmic localization. We detected a higher amount of TDP-43 in epidermis (*p* < 0.001) and in both layers of dermis (*p* < 0.001), as well as a higher percentage of TDP-43 cytoplasmic positive cells (*p* < 0.001) in the ALS group compared to HC and NC groups. Dermal cells containing TDP-43 were fibroblasts as identified by co-labeling against vimentin. ROC analyses (AUC 0.867, *p* < 0.001; CI 95% 0.800–0.935) showed that detection of 24.1% cells with cytoplasmic TDP-43 positivity in the dermis had 85% sensitivity and 80% specificity for detecting ALS. We have identified significantly increased TDP-43 levels in epidermis and in the cytoplasm of dermal cells of ALS patients. Our findings provide support for the use of TDP-43 in skin biopsies as a potential biomarker.

## 1. Introduction

Amyotrophic lateral sclerosis (ALS) is a neurodegenerative disorder affecting motor neurons from cortex, brainstem, and spinal cord. While most are sporadic cases, 10–15% are familial forms [1,2]. Studies derived from the genetic forms have expanded the spectrum of the disease to extra-motor manifestations such as cognitive impairment, extrapyramidal or neuropsychiatric symptoms [3,4,5,6]. Diagnosis is based on clinical symptoms and often is established relatively late; therefore, there is a need to identify biomarkers for the early diagnosis of ALS that could allow for monitoring disease progression and to start early neuroprotective treatment to prevent the motoneuron degeneration. The 43-kDa TAR DNA-binding protein (TDP-43) is a ubiquitous DNA binding protein with multiple functions encoded by the TARDBP gene, and its mutations have been associated with autosomal dominant ALS and frontotemporal dementia (FTD) [7,8]. However, the more than 50 missense mutations identified in TARDBP only account for 1–2% of total ALS cases [9]. Nevertheless, the importance of TDP-43 in ALS relies on the fact that it is a major component of the ubiquitinated insoluble cytoplasmic inclusions, concomitant with a loss of nuclear TDP-43 in upper and lower motor neurons and in other regions of the central nervous system in most patients (both sporadic and familial, with or without TARDBP mutations) [10]. These inclusions are widespread, regardless of the location of symptoms onset, and considered a pathological hallmark of ALS-FTD spectrum [11,12]. In healthy motoneurons, the TDP-43 protein is located in the nucleus, suggesting that the increased TDP-43 cytoplasmic translocation may be a potential pathological biomarker. However, monitoring this alteration using biopsies from the central nervous system is unpractical. Nevertheless, alterations in the skin and other tissues may precede or appear concomitantly with neurological symptoms in some neurodegenerative diseases [13]. Despite the variability among the studies performed on either skin biopsies, cultured fibroblasts or engineered skin tissue, there is a common finding of TDP-43 cytoplasmic accumulation in the skin of sporadic and familial ALS patients [14,15,16,17,18,19,20,21]. Nonetheless, there is not a well-defined relationship with disease progression and other clinical features. Moreover, nuclear TDP-43 expression is found in skin cells, fibroblasts, keratinocytes, Langerhans cells and melanocytes of healthy individuals, but importantly, much lower amounts are found in the cytoplasm [22,23]. Therefore, although cutaneous TDP-43 seems a promising candidate as a minimally invasive biomarker of the disease, its role is still undefined [22]. Quantitative detailed studies are needed to define the role of skin TDP-43 and its relationship with clinical features. The objective of this study was to further investigate the cytoplasmic localization of TDP-43 in the skin of ALS patients, quantifying its accumulation to identify more accessible histopathological hallmarks of ALS. Our results show an increase in TDP-43 in ALS patients in both epidermis and dermis, establishing a proof-of-concept of its possible value in the diagnosis of this pathology.

## 2. Materials and Methods

### 2.1. Participants and Samples

Forty-four subjects with definite ALS and 10 healthy controls (HC) were prospectively recruited by the ALS units from Hospital del Mar and Hospital de Bellvitge, Barcelona. A third group of 10 neurological controls (NC) were also selected. ALS patients fulfilled the diagnostic criteria (revised El Escorial criteria) [24] and were selected by experienced neurologists specialized in motor neuron diseases. ALS patients were 22 females and 22 males, with a median age of 66 years (IQR 58–73). Healthy subjects did not have any signs, symptoms, or history of neurological diseases; 6 were female, with a median age of 59 years (IQR 43–62). The NC group consisted of 5 patients with Parkinson disease and 5 with relapsing-remitting multiple sclerosis, 4 of them were female, and had a median age of 59 years (IQR 54–66). There were no statistically significant differences in sociodemographic variables between the groups. Clinical data such as site of onset, time from onset of the disease to the biopsy and ALSFRS-R slope were obtained for the ALS group. Of the ALS patients, 18 (40.9%) had spinal onset and 26 bulbar onset (59.1%), and the median ALSFR-R slope at the time of the skin biopsy was 1.29 (IQR 0.63–2.18). Median time from onset of ALS symptoms to biopsy was 11.5 months (IQR 8.52–19.13). All ALS patients were tested for the hexanucleotide expansion in the C9ORF72 gene, and in all but one, no pathological expansion was detected. The study was approved by the local ethics committee of both participating hospitals. Written informed consent for skin biopsy was obtained from all subjects. Any method detail and data not published within the article will be shared anonymized by reasonable request from any qualified investigator. The samples were shipped to our laboratory coded, and the experimenters performing processing and immunohistochemical analyses were blind with respect to the diagnosis.

### 2.2. Skin Biopsy and Processing

Three-millimeter skin punch biopsies were obtained from the distal leg after local anesthesia with mepivacaine. Skin biopsies were fixed in paraformaldehyde solution at 4% and maintained at 4 °C in PB (phosphate buffer) with sucrose. Then, biopsies were cut by cryotome into 60 µm thick sections collecting the slices in PBS.

For immunofluorescence, sections were blocked with endogenous Biotin-Blocking Kit (Invitrogen), TBS-Triton 0.3% and normal donkey serum 10%, and subsequently incubated overnight at 4 °C with anti-TDP-43 antibody (1:200, cat#12892-1-AP, Proteintech, Manchester, UK), anti-vimentin (1:200, Sigma, St. Louis, MO, USA) and rabbit anti-protein gene product 9.5 (PGP9.5, 1:50, Cedarlane, Burlington, Canada) as primary antibodies. After washes, sections were incubated overnight at 4 °C with horse anti-rabbit biotinylated antibody (1:200, Vector Laboratories, Burlingame, California, USA), conjugated streptavidin Alexa Fluor 488 (1:200, Thermo Fisher, Waltham, Massachusetts, USA) or streptavidin Alexa Fluor 594 (1:200, Thermo Fisher, Waltham, Massachusetts, USA) and donkey anti-rabbit cyanine 3 (1:200, Jackson Immunoresearch, Ely, Cambridgeshire, UK) as secondary antibodies. Slices were then transferred to gelatinized slides and mounted in Fluoromount G (SouthernBiotech, Birmingham, AL, USA). To assess antibody specificity, control samples were processed in parallel as described before but without primary antibodies. DAPI staining was used to ensure correct recognition of tissue structures. The anti-TDP43 antibody used in this study recognizes the C terminal cleavage product (20–30 KDa) and the native and phosphorylated forms of TDP-43. Antibodies against the non-phosphorylated form have been widely used to determine the TDP-43 translocation to the cytoplasm without the risk of missing different post-translational modifications and non-phosphorylated cytosolic distribution of TDP-43 [12,16,25]. In the studies published to date on skin of ALS patients, only two have used antibodies against *p*-TDP-43, finding no labeling in one of them [26] and labeling only one-third of the cases in the other [27]. The rest of the studies have successfully used antibodies against the non-phosphorylated form [14,15,16,17,18,19,20,21]. 

### 2.3. Confocal Imaging and Measurements

**Figure 1:**
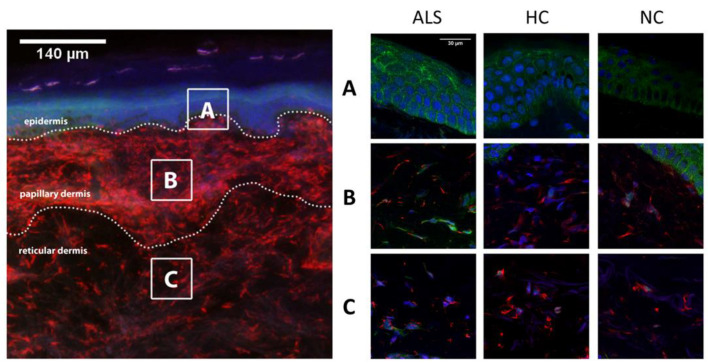
Cytoplasmic TDP-43 immunoreactivity was analyzed in at least two areas of epidermis, papillary and reticular dermis (dotted rectangular area), per case (left panel). In the right panel, representative confocal microscopy images of epidermis (**A**), papillary dermis (**B**) and reticular dermis (**C**) from ALS, HC and NC subjects are shown. TDP-43 was found profusely in epidermis in all cases, although with higher density in the ALS group (*p* < 0.001) compared to both HC and NC groups. In the dermis of HC and NC subjects, TDP-43 was mostly seen in the nucleus, whereas cytoplasmic aggregations were more present in ALS cases in papillary and reticular dermis (*p* < 0.001). ALS: amyotrophic lateral sclerosis; HC: healthy controls; NC: neurological controls.

Immunolabeled sections were first viewed under an Olympus BX-51 microscope equipped for epifluorescence using appropriate filters. Areas of interest were analyzed with a scanning confocal microscope (Figure 1).

Epidermal TDP-43 amount was measured in microphotographs taken from two representative areas of epidermis (101.61 × 101.61 µm^2^ each) of each case, using ImageJ software (version 2.1.0/1.53c, Wayne Rasband, Bethesda, Maryland, USA). The mean of the two measures of the percentage of area with TDP-43 labeling was calculated after defining the threshold background correction. 

The two layers of the dermis (papillary and reticular dermis) were evaluated separately. This distinction was made given that papillary and reticular cells have different patterns of protein synthesis and expression [25,28]. Differentiation of both layers was based on visual recognition of different density of connective tissue, and the superficial vascular plexus at the boundaries between both layers. Images of two representative areas (101.61 × 101.61 µm^2^ each) of papillary and reticular dermis were taken and analyzed. The representative areas were selected in a systematic way, avoiding areas of the section that might have artefacts and then selecting 2 evenly separated fields (for epidermis, papillary and reticular dermis) at low magnification, and then viewing them under higher magnification for capture and analysis. The levels of cytoplasmic TDP-43 were quantified as (1) percentage of cytoplasmic TDP-43 immunoreactivity and (2) percentage of cells with positive TDP-43 into the cytoplasm in each defined area. For calculating the percentage of cytoplasmic TDP43 immunoreactivity, first, a threshold was defined for background correction, then the percentage of pixels in the area above threshold of TDP-43 labeling was measured using ImageJ software. Additionally, the mean percentage of cells with TDP-43 positivity within their cytoplasm was calculated in the analyzed confocal images, relative to the total number of vimentin positive cells (fibroblasts) in these areas. Results are expressed as the mean of the two measures per subject and layer.

### 2.4. Intraepidermal Innervation and TDP-43 Nerve Colocalization

To study the possible colocalization of TDP-43 in the cutaneous innervation, we performed immunohistochemical co-labeling for PGP9.5 and TDP-43. Confocal images were taken with a confocal microscope for counting intraepidermal nerve fibers (IENF) and for TDP-43 colocalization assessment. IENF were measured in a subset of ALS and HC subjects. Individual fibers were counted as they pass through the basement membrane, whereas branching occurring within the epidermis did not increase the number of the IENFs counted. IENF density was expressed as the number of fibers per 1 mm length of the epidermis. Average density of IENFs in two images per sample was then derived. 

### 2.5. Data Analysis

Data are expressed as mean ± SEM. Means were compared by ANOVA, applying Tukey’s post hoc test when necessary (SPSS statistics 19 software, IBM, Armonk, NY, USA). The level of significance was set at *p* < 0.05. Pearson’s correlation coefficient was used to assess possible linear association between two continuous quantitative variables. In order to obtain an estimate of sensitivity and specificity of TDP-43 quantification regarding ALS diagnosis, ROC curves and area under the curve (AUC) were calculated and optimal cut-off values were selected using Youden’s index. 

## 3. Results

### 3.1. TDP-43 Cytoplasmic Localization in ALS Patients Skin Biopsies

To determine cytoplasmic TDP-43 accumulation, a signature of ALS pathology, skin biopsies, were labeled with polyclonal antibody against TDP-43. The results of TDP-43 immunoreactivity levels and proportion of cells with cytoplasmic TDP-43 positivity in epidermis, papillary and reticular dermis are summarized in Table 1.

**Figure 2:**
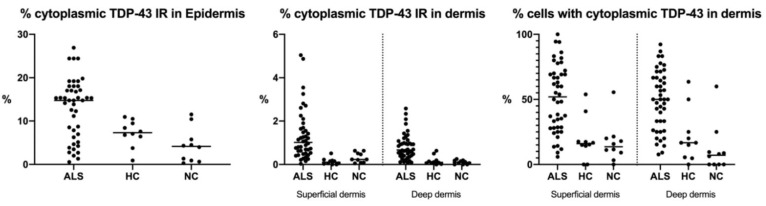
Distribution of the amount of cytoplasmic TDP-43 content and cells with cytoplasmic TDP-43 of the three cohorts in the different skin layers. IR: immunoreactivity; ALS: amyotrophic lateral sclerosis; HC: healthy controls; NC: neurological controls.

In epidermis, TDP-43 was distributed in stratum basale and stratum spinosum. Absence of TDP-43 in the outer layers of the skin may be explained by the process of terminal differentiation of keratinocytes in granulosum and corneum strata. We detected a higher expression of TDP-43 in epidermal cells (*p* < 0.001) in the ALS group (12.9 ± 1.0%) compared to HC (7.2 ± 1.0%) and NC (4.4 ± 1.2%) (Figure 2).

**Figure 3:**
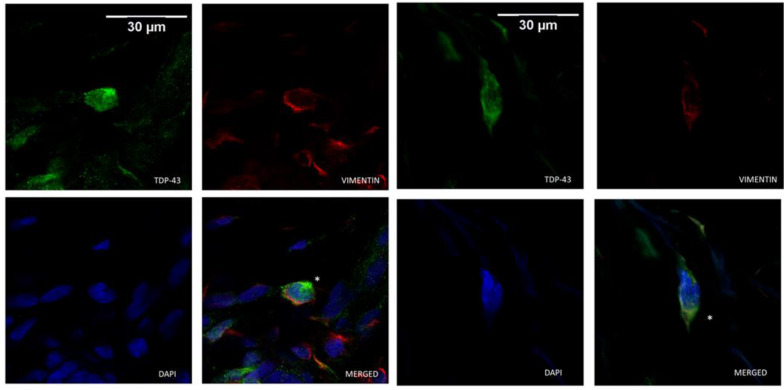
Cytoplasmic TDP-43-positive cells (*) were identified as fibroblasts based on vimentin immunoreactivity. Representative confocal microscopy images of ALS dermal fibroblasts co-immunolabeled for TDP-43 (in green), vimentin (in red) and DAPI nuclear staining (in blue).

Cytoplasmic TDP-43 was detected in dermal cells of ALS patients and controls. However, a higher number of positive cells for cytosolic labeling of TDP-43 was found in ALS patients than in healthy and NC. Those dermal cells were identified as fibroblasts as they were positively marked with vimentin (Figure 3). Unlike what was seen in the epidermis, fibroblasts expressing TDP-43 were not widely distributed, and represented only a small percentage of dermal cells. In dermal cells, TDP-43 cytoplasmic distribution varied from small amounts around the nucleus to a more scattered deposition, whereas in the epidermis, those aggregates were more dense and evenly diffused within the cells. The immunoreactivity against TDP-43 as well as the percentage of fibroblast cells with cytoplasmic label for TDP-43 were significantly increased in the ALS group compared with the HC and NC groups in both the papillary and reticular dermis (Table 1). 

**Figure 4:**
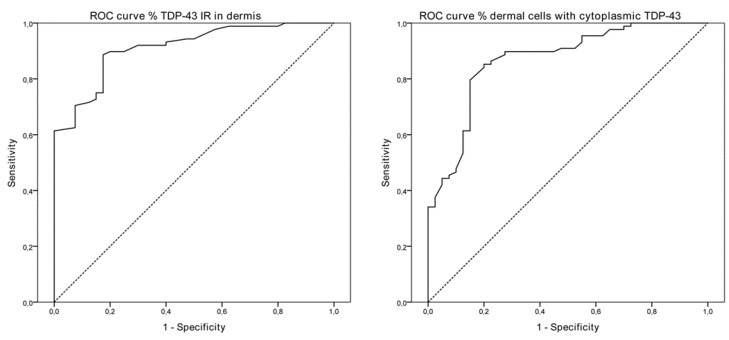
ROC curves of the percentage of TDP-43 immunoreactivity in both layers of the dermis (AUC 0.911, CI 95% 0.862–0.960, *p* < 0.001) and of the percentage of dermal cells with cytoplasmic TDP-43 labeling (AUC 0.867, CI 95% 0.800–0.935, *p* < 0.001) in ALS patients. ROC: receiver operation characteristic; AUC: area under the ROC curve.

We performed an ROC analysis based on the percentage of TDP-43 cytoplasmic-positive cells of the overall dermis as well as on the percentage of immunoreactivity to achieve a theoretical cut-off point as a proof of concept of its value as a marker of the disease. ROC curves and AUC (0.867, *p* < 0.001; CI 95% 0.800–0.935) were calculated for TDP-43-positive dermal cells, including measures from superficial/deep dermis, and cut-off values were selected according to optimal Youden’s index. Detection of 24.14% of cells with cytoplasmic TDP-43 positivity in the dermis had 85.2% sensitivity and 80.0% specificity, with a positive predictive value of 90.4% and a negative predictive value of 71.1% (Figure 4). Considering the proportion of TDP-43 immunoreactivity in the dermis, ROC analyses showed an AUC of 0.911 (*p* < 0.001, CI 95% 0.862–0.960) and cut-off values of 0.26%, with 88.6% sensitivity, 82.5% specificity, 91.8% positive predictive value and 76.7% negative predictive value for detecting ALS. 

There were no differences in TDP-43 levels (immunoreactivity and percentage of cells with cytoplasmic TDP-43 positivity) in epidermis and papillary dermis according to gender and site of onset in ALS patients. Only in the reticular dermis we found a significant lower TDP-43 immunoreactivity in bulbar onset patients, but no differences were found considering the percentage of cells with cytoplasmic TDP-43 localization. No significant correlations were found between TDP-43 expression measurements and age of onset of the disease, time from symptoms onset to biopsy and ALSFRS-R slope (Table 2; Supplemental Figure S1).

### 3.2. TDP-43 Changes over Time

A small subset of ALS patients (eight in total; three bulbar and five spinal onset) underwent a second skin biopsy 12 months later. There were no significant differences in levels of TDP-43 immunoreactivity in epidermis (17.29 ± 5.33 vs. 18.58 ± 3.68; *p* = 0.831), superficial dermis (1.17 ± 0.34 vs. 8.58 ± 6.24; *p* = 0.283) and deep dermis (3.85 ± 3.17 vs. 16.29 ± 11.32; *p* = 0.336) between the initial biopsy and 12 months later. The increase in levels of TDP-43 in the dermis at the second biopsy did not achieve statistical significance due to the low number of samples analyzed. However, the percentage of cells with cytoplasmic TDP-43 translocation in the dermis increased in the second biopsy, with differences being significant for the papillary dermis (34.82 ± 7.79 vs. 62.13 ± 3.82; *p* = 0.034), but not for the reticular dermis (49.73 ± 10.01 vs. 62.38 ± 2.32; *p* = 0.241).

### 3.3. TDP-43 and Epidermal Nerve Fibers

**Figure 5:**
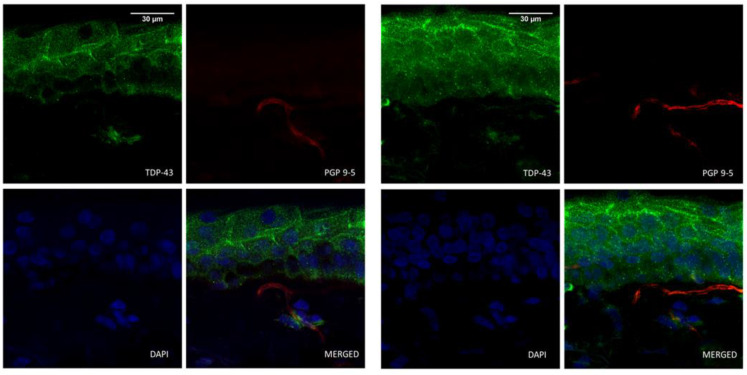
Representative images of ALS skin sections immunohistochemically labeled for PGP 9.5 (in red) to identify the innervation and for TDP-43 (in green). In the images, subepidermal nerve plexus and intraepidermal nerve fibers are shown with no colocalization of TDP-43 immunoreactivity.

We analyzed the IENF density of 18 ALS patients and six controls. The ALS group presented lower values of IENF (7.78 ± 0.80; *p* = 0.010) compared with HC (12.75 ± 2.20) and NC (10.89 ± 1.77). However, no colocalization of TDP-43 was found in PGP9.5-labeled fibers in ALS patients, HC or NC (Figure 5).

## 4. Discussion

The results of this study show that cytosolic localization of TDP-43 is increased in the skin cells of ALS patients and can be detected by immunofluorescence of skin biopsies. TDP-43 presence in the cytoplasm of dermal fibroblasts is particularly relevant and represents a potentially useful biomarker of ALS.

TDP-43 cytoplasmic deposition and its clearance from the nucleus have been observed beyond motoneurons in other areas of the nervous system, and in extra-neural tissues [19,29,30,31,32,33]. However, few studies have explored the presence of TPD-43 protein in skin of ALS patients, and quantitative information from the dermis of skin biopsies was missing. Suzuki et al. [14] analyzed skin biopsies from 15 ALS patients and 15 neurological controls and found higher amounts of TDP-43 in the ALS group, although they did not report on the nuclear/cytoplasmic localization of the protein. They also observed TDP-43 immunoreactivity in both neurological controls and ALS in blood vessels and glands, but it was lower in the case of controls [30]. In a tissue-engineered model derived from fibroblasts of 12 ALS patients (six sporadic and six familial C9ORF72 cases), an increased accumulation of cytoplasmic TDP-43 was found in ALS patients (30%) compared to HC (4%) in epidermis, dermo-epidermal junction, and dermis [14]. Wang et al. [15] observed in patients harboring the TARDBP A315T mutation that the presence of cytoplasmic TDP-43 occurs mainly in epidermis but also in dermis, although no quantification was performed. Another study with skin biopsies from 22 ALS patients and 26 neurological controls (neuropathies) [19] analyzed TDP-43 mRNA expression, epidermal TDP-43 immunoblot and percentage of TDP-43-positive cells in epidermis. They did not find differences in the immunohistochemical analysis, although differences in immunoblot TDP-43 expression with a reduction of mRNA in ALS epidermis suggested a dysregulation of protein expression.

Studies on cultured fibroblasts have shown abnormal TDP-43 cytoplasmic aggregations in ALS (sporadic and familial cases of SOD1, TARDBP, FUS and C9ORF72 mutations) [17], even with different patterns regarding nuclear/cytoplasmic deposition ratio between patients harboring different mutations. Other studies have focused on the cytoplasmic aggregation in cultured fibroblasts under the presence of stress in both sporadic and familial cases (UBQLN2, UBQLN1, TARDBP) [18,20,21]. Only one study to date based on cultured fibroblasts from sporadic ALS patients did not detect any TDP-43 accumulation [26]. Authors speculated that methodological aspects could explain differences from other similar studies.

TDP-43 accumulation is not homogenous throughout the motor nervous system of ALS patients [12]. Full-length protein can be found in both cortical and spinal motor neurons, but C-terminal fragments have only been observed in brain neurons [34,35]. These differences in affected neurons raise the question if TDP-43 proteinopathy may be heterogeneous also in non-neuronal cells [36]. As expected, based on previous observations [37], we found a lower IENF density in the ALS group, but we did not find any colocalization of TDP-43 in dermal or intraepidermal nerve fibers. To date, only deposition of TDP-43 in autonomic dermal fibers has been shown [27].

We did not find a correlation between TDP-43 deposition and clinical data. There are only few previous reports about clinical correlation, and only disease duration was analyzed with contradictory results. In the subgroup of patients with a second biopsy 12 months later, there was a high variability of TDP-43 immunoreactivity between the first and second biopsies. Although changes were not significant, a higher number of dermal cells with cytoplasmic TDP-43 deposition were detected in the superficial dermis. However, the conclusions from this subgroup should be taken with caution due to the small number of patients, and the limited time interval between biopsies in a fast-progression disease such as ALS.

Albeit having some distinctive histopathological features, ALS lacks an accessible biomarker. Diagnosis is based on clinical criteria and supported by neurophysiological studies [24,38,39]. Although these criteria have been revised to achieve an early diagnosis with proper sensitivity and specificity, the time from symptoms onset to diagnosis ranges from 8 to 16 months, and even a non-negligible proportion of patients die without achieving a sufficient diagnostic certainty [40,41]. Even in the absence of a curative treatment, the benefits of a biomarker should not be overlooked, whether it is for the possibility of an earlier diagnosis in suspected cases, presymptomatic detection and an optimal selection of candidates for clinical trials. For this reason, determination of TDP-43 in corporal fluids or tissue as a potential biomarker has been pursued [42]. Different approaches have been taken for the detection and quantification of TDP-43 in blood [43,44,45] and cerebrospinal fluid (CSF) in ALS and FTD [46,47,48,49]. However, the ubiquitous nature of the TDP-43 protein makes its detection and interpretation more difficult compared to the detection of amyloid or tau (purely neuronal proteins) in Alzheimer’s disease [50].

TDP-43 cytoplasmic localization is present in epidermal keratinocytes and in dermal fibroblasts of ALS and HC, but quantitative analyses show significantly larger amounts in ALS patients. Quantitative values of dermal cells with TDP-43 appear to offer a sensitive and minimally invasive biomarker. Further experiments are needed to specifically explore the levels of post-translational modifications of TDP-43 in these samples, such as phosphorylation, ubiquitylation and truncated forms. Although the number of skin samples from ALS patients represents the larger series reported to date, a higher amount is needed to achieve practical cut-off values of TDP-43 deposition for diagnostic purposes. Nevertheless, even considering the limitations, this work represents the proof of concept of cutaneous TDP-43 as a potential biomarker for ALS.

## Figures and Tables

**Table 1:** Results of TDP-43 immunoreactivity and cells with cytoplasmic TDP-43-positive cells. Values are shown as mean and SEM. ALS: amyotrophic lateral sclerosis; HC: healthy controls; NC: neurological controls (5 multiple sclerosis and 5 Parkinson’s disease); IR: immunoreactivity.

	ALS (N = 44)	HC (N = 10)	NC (N = 10)	*p* Value
**Epidermis**				
% TDP-43 IR	12.94 ± 1.05	7.19 ± 0.97	4.37 ± 1.25	<0.001
**Papillary Dermis**				
% TDP-43 IR	1.33 ± 0.17	0.13 ± 0.05	0.31 ± 0.07	<0.001
% Cytoplasmic TDP-43^+^ cells	50.88 ± 3.91	19.11 ± 5.2	16.58 ± 4.85	<0.001
**Reticular Dermis**				
% TDP-43 IR	0.82 ± 0.09	0.18 ± 0.07	0.10 ± 0.03	<0.001
% Cytoplasmic TDP-43^+^ cells	50.36 ± 3.64	21.73 ± 6.40	11.75 ± 5.88	<0.001

**Table 2:** TDP-43 quantification and clinical features of ALS patients (*N* = 44). IR: immunoreactivity; R: Pearson correlation coefficient.

	Epidermis	Superficial Dermis		Deep Dermis	
	% TDP-43 IR	% TDP-43 IR	% Cytoplasmic TDP-43^+^ Cells	% TDP-43 IR	% Cytoplasmic TDP-43^+^ Cells
**Gender**					
male female	14.66 ± 1.33	1.24 ± 0.19	47.37 ± 5.60	0.85 ± 0.12	50.96 ± 5.34
	11.21 ± 1.57	1.43 ± 0.29	54.39 ± 5.49	0.81 ± 0.13	47.95 ± 4.50
	*p* = 0.102	*p* = 0.601	*p* = 0.376	*p* = 0.832	*p* = 0.669
**Site of onset**					
spinal bulbar	14.31 ± 1.21	1.33 ± 0.20	46.57 ± 5.30	0.98 ± 0.12	49.02 ± 4.40
	10.52 ± 1.86	1.34 ± 0.34	58.43 ± 5.12	0.55 ± 0.08	50.21 ± 5.76
	*p* = 0.083	*p* = 0.991	*p* = 0.147	*p* = 0.017	*p* = 0.871
**Age**	R = −0.050	R = −0.180	R = −0.199	R = 0.043	R = −0.114
	*p* = 0.747	*p* = 0.243	*p* = 0.194	*p* = 0.780	*p* = 0.460
**Time from onset to biopsy**	R = 0.071	R = 0.032	R = −0.115	R = 0.053	R = −0.119
	*p* = 0.646	*p* = 0.836	*p* = 0.457	*p* = 0.731	*p* = 0.440
**ALSFRS-R slope**	R = −0.169	R = −0.205	R = −0.046	R = −0.086	R = 0.057
	*p* = 0.277	*p* = 0.186	*p* = 0.772	*p* = 0.585	*p* = 0.717

## Data Availability

The data presented in this study are available on request from the corresponding author.

## Supplementary Materials

The following supporting information can be downloaded at: https://www.mdpi.com/article/10.3390/cells11020209/s1, Figure S1: Correlation graphs of the percentage of cytoplasmic TDP-43 immunoreactivity with age, time from onset to biopsy and ALSFRS-R slope in the three different skin layers.

## References

[1] Hardiman O., Al-Chalabi A., Chio A., Corr E.M., Logroscino G., Robberecht W., Van Den Berg L.H. (2017). Amyotrophic lateral sclerosis. Nat. Rev. Dis. Primers.

[2] Mancuso R., Navarro X. (2015). Amyotrophic lateral sclerosis: Current perspectives from basic research to the clinic. Prog. Neurobiol..

[3] McCombe P.A., Wray N.R., Henderson R.D. (2017). Extra-motor abnormalities in amyotrophic lateral sclerosis: Another layer of heterogeneity. Expert Rev. Neurother..

[4] McCluskey L., Vandriel S., Elman L., Van Deerlin V.M., Powers J., Boller A., Wood E.M., Woo J., McMillan C.T., Rascovsky K. (2014). ALS-Plus syndrome: Non-pyramidal features in a large ALS cohort. J. Neurol. Sci..

[5] Strong M.J. (2008). The syndromes of frontotemporal dysfunction in amyotrophic lateral sclerosis. Amyotroph. Lateral Scler..

[6] Phukan J., Pender N., Hardiman O. (2007). Cognitive impairment in amyotrophic lateral sclerosis. Lancet Neurol..

[7] Kwong L.K., Neumann M., Sampathu D.M., Lee V.M.-Y., Trojanowski J.Q. (2007). TDP-43 proteinopathy: The neuropathology underlying major forms of sporadic and familial frontotemporal lobar degeneration and motor neuron disease. Acta Neuropathol..

[8] Neumann M., Sampathu D.M., Kwong L.K., Truax A.C., Micsenyi M.C., Chou T.T., Bruce J., Schuck T., Grossman M., Clark C.M. (2006). Ubiquitinated TDP-43 in Frontotemporal Lobar Degeneration and Amyotrophic Lateral Sclerosis. Science.

[9] Chia R., Chiò A., Traynor B.J. (2018). Novel genes associated with amyotrophic lateral sclerosis: Diagnostic and clinical implications. Lancet Neurol..

[10] Brettschneider J., Del Tredici K., Toledo J.B., Robinson J.L., Irwin D.J., Grossman M., Suh E.R., Van Deerlin V.M., Wood E.M., Baek Y. (2013). Stages of pTDP-43 pathology in amyotrophic lateral sclerosis. Ann. Neurol..

[11] Neumann M. (2009). Molecular Neuropathology of TDP-43 Proteinopathies. Int. J. Mol. Sci..

[12] Braak H., Ludolph A.C., Neumann M., Ravits J., Del Tredici K. (2017). Pathological TDP-43 changes in Betz cells differ from those in bulbar and spinal α-motoneurons in sporadic amyotrophic lateral sclerosis. Acta Neuropathol..

[13] Clos A.L., Kayed R., Lasagna-Reeves C.A. (2012). Association of Skin with the Pathogenesis and Treatment of Neurodegenerative Amyloidosis. Front. Neurol..

[14] Suzuki M., Mikami H., Watanabe T., Yamano T., Yamazaki T., Nomura M., Yasui K., Ishikawa H., Ono S. (2010). Increased expression of TDP-43 in the skin of amyotrophic lateral sclerosis. Acta Neurol. Scand..

[15] Wang X., Zhou S., Ding X., Ma M., Zhang J., Zhou Y., Wu E., Teng J. (2015). Activation of ER Stress and Autophagy Induced by TDP-43 A315T as Pathogenic Mechanism and the Corresponding Histological Changes in Skin as Potential Biomarker for ALS with the Mutation. Int. J. Biol. Sci..

[16] Paré B., Touzel-Deschênes L., Lamontagne R., Lamarre M.-S., Scott F.-D., Khuong H.T., Dion P.A., Bouchard J.-P., Gould P., Rouleau G.A. (2015). Early detection of structural abnormalities and cytoplasmic accumulation of TDP-43 in tissue-engineered skins derived from ALS patients. Acta Neuropathol. Commun..

[17] Sabatelli M., Zollino M., Conte A., Del Grande A., Marangi G., Lucchini M., Mirabella M., Romano A., Piacentini R., Bisogni G. (2015). Primary fibroblasts cultures reveal TDP-43 abnormalities in amyotrophic lateral sclerosis patients with and without SOD1 mutations. Neurobiol. Aging.

[18] Yang S., Zhang K.Y., Kariawasam R., Bax M., Fifita J.A., Ooi L., Yerbury J.J., Nicholson G.A., Blair I.P. (2015). Evaluation of Skin Fibroblasts from Amyotrophic Lateral Sclerosis Patients for the Rapid Study of Pathological Features. Neurotox. Res..

[19] Abe K., Ohkubo T., Yokota T. (2017). TDP-43 in the skin of amyotrophic lateral sclerosis patients. J. Med. Dent. Sci..

[20] Riancho J., Castanedo-Vázquez D., Gil-Bea F., Tapia O., Arozamena J., Durán-Vían C., Sedano M.J., Berciano M.T., De Munain A.L., Lafarga M. (2020). ALS-derived fibroblasts exhibit reduced proliferation rate, cytoplasmic TDP-43 aggregation and a higher susceptibility to DNA damage. J. Neurol..

[21] Romano N., Catalani A., Lattante S., Belardo A., Proietti S., Bertini L., Ceci M. (2020). ALS skin fibroblasts reveal oxidative stress and ERK1/2-mediated cytoplasmic localization of TDP-43. Cell. Signal..

[22] Uhlen M., Oksvold P., Fagerberg L., Lundberg E., Jonasson K., Forsberg M., Zwahlen M., Kampf C., Wester K., Hober S. (2010). Towards a knowledge-based Human Protein Atlas. Nat. Biotechnol..

[23] Thul P.J., Åkesson L., Wiking M., Mahdessian D., Geladaki A., Blal H.A., Alm T., Asplund A., Björk L., Breckels L.M. (2017). A subcellular map of the human proteome. Science.

[24] Brooks B.R., Miller R.G., Swash M., Munsat T.L. (2000). El Escorial revisited: Revised criteria for the diagnosis of amyotrophic lateral sclerosis. Amyotroph. Lateral Scler. Other Mot. Neuron. Disord..

[25] Williams S.M., Khan G., Harris B.T., Ravits J., Sierks M.R. (2017). TDP-43 protein variants as biomarkers in amyotrophic lateral sclerosis. BMC Neurosci..

[26] Codron P., Cassereau J., Vourc’H P., Veyrat-Durebex C., Blasco H., Kane S., Procaccio V., Letournel F., Verny C., Lenaers G. (2018). Primary fibroblasts derived from sporadic amyotrophic lateral sclerosis patients do not show ALS cytological lesions. Amyotroph. Lateral Scler. Front. Degener..

[27] Ren Y., Liu W., Li Y., Sun B., Li Y., Yang F., Wang H., Li M., Cui F., Huang X. (2018). Cutaneous somatic and autonomic nerve TDP-43 deposition in amyotrophic lateral sclerosis. J. Neurol..

[28] Ono S. (2000). The skin in amyotrophic lateral sclerosis. Amyotroph. Lateral Scler. Other Mot. Neuron Disord..

[29] Zhang H., Tan C.-F., Mori F., Tanji K., Kakita A., Takahashi H., Wakabayashi K. (2007). TDP-43-immunoreactive neuronal and glial inclusions in the neostriatum in amyotrophic lateral sclerosis with and without dementia. Acta Neuropathol..

[30] Paré B., Gros-Louis F. (2017). Potential skin involvement in ALS: Revisiting Charcot’s observation–a review of skin abnormalities in ALS. Rev. Neurosci..

[31] Neumann M., Kwong L.K., Lee E.B., Kremmer E., Flatley A., Xu Y., Lee V.M.Y. (2009). Phosphorylation of S409/410 of TDP-43 is a consistent feature in all sporadic and familial forms of TDP-43 proteinopathies. Acta Neuropathol..

[32] Hasegawa M., Arai T., Nonaka T., Kametani F., Yoshida M., Hashizume Y., Beach T.G., Buratti E., Baralle F., Morita M. (2008). Phosphorylated TDP-43 in frontotemporal lobar degeneration and amyotrophic lateral sclerosis. Ann. Neurol..

[33] Miki Y., Mori F., Nunomura J., Ookawa K., Yajima N., Yagihashi S., Wakabayashi K. (2010). Sporadic amyotrophic lateral sclerosis with pallido-nigro-luysian degeneration: A TDP-43 immunohistochemical study. Neuropathology.

[34] Igaz L.M., Kwong L.K., Xu Y., Truax A.C., Uryu K., Neumann M., Clark C.M., Elman L.B., Miller B.L., Grossman M. (2008). Enrichment of C-Terminal Fragments in TAR DNA-Binding Protein-43 Cytoplasmic Inclusions in Brain but not in Spinal Cord of Frontotemporal Lobar Degeneration and Amyotrophic Lateral Sclerosis. Am. J. Pathol..

[35] Nishihira Y., Tan C.-F., Onodera O., Toyoshima Y., Yamada M., Morita T., Nishizawa M., Kakita A., Takahashi H. (2008). Sporadic amyotrophic lateral sclerosis: Two pathological patterns shown by analysis of distribution of TDP-43-immunoreactive neuronal and glial cytoplasmic inclusions. Acta Neuropathol..

[36] Weskamp K., Tank E.M., Miguez R., McBride J.P., Gómez N.B., White M., Lin Z., Gonzalez C.M., Serio A., Sreedharan J. (2020). Shortened TDP43 isoforms upregulated by neuronal hyperactivity drive TDP43 pathology in ALS. J. Clin. Investig..

[37] Weis J., Katona I., Muller-Newen G., Sommer C., Necula G., Hendrich C., Ludolph A.C., Sperfeld A.-D. (2011). Small-fiber neuropathy in patients with ALS. Neurology.

[38] de Carvalho M., Dengler R., Eisen A., England J.D., Kaji R., Kimura J., Swash M. (2008). Electrodiagnostic criteria for diagnosis of ALS. Clin. Neurophysiol..

[39] Shefner J.M., Al-Chalabi A., Baker M.R., Cui L.-Y., de Carvalho M., Eisen A., Grosskreutz J., Hardiman O., Henderson R., Matamala J.M. (2020). A proposal for new diagnostic criteria for ALS. Clin. Neurophysiol..

[40] Palese F., Sartori A., Logroscino G., Pisa F.E. (2019). Predictors of diagnostic delay in amyotrophic lateral sclerosis: A cohort study based on administrative and electronic medical records data. Amyotroph. Lateral Scler. Front. Degener..

[41] Paganoni S., Macklin E., Lee A., Murphy A., Chang J., Zipf A., Cudkowicz M., Atassi N. (2014). Diagnostic timelines and delays in diagnosing amyotrophic lateral sclerosis (ALS). Amyotroph. Lateral Scler. Front. Degener..

[42] Feneberg E., Gray E., Ansorge O., Talbot K., Turner M. (2018). Towards a TDP-43-Based Biomarker for ALS and FTLD. Mol. Neurobiol..

[43] Verstraete E., Kuiperij H.B., Van Blitterswijk M.M., Veldink J.H., Schelhaas H.J., Van Den Berg L.H., Verbeek M.M. (2012). TDP-43 plasma levels are higher in amyotrophic lateral sclerosis. Amyotroph. Lateral Scler..

[44] De Marco G., Lomartire A., Calvo A., Risso A., De Luca E., Mostert M., Chiò A. (2017). Monocytes of patients with amyotrophic lateral sclerosis linked to gene mutations display altered TDP-43 subcellular distribution. Neuropathol. Appl. Neurobiol..

[45] De Marco G., Lupino E., Calvo A., Moglia C., Buccinna B., Grifoni S., Chio A. (2011). Cytoplasmic accumulation of TDP-43 in circulating lymphomonocytes of ALS patients with and without TARDBP mutations. Acta Neuropathol..

[46] Junttila A., Kuvaja M., Hartikainen P., Siloaho M., Helisalmi S., Moilanen V., Kiviharju A., Jansson L., Tienari P.J., Remes A.M. (2016). Cerebrospinal Fluid TDP-43 in Frontotemporal Lobar Degeneration and Amyotrophic Lateral Sclerosis Patients with and without the C9ORF72 Hexanucleotide Expansion. Dement. Geriatr. Cogn. Disord. Extra.

[47] Noto Y.-I., Shibuya K., Sato Y., Kanai K., Misawa S., Sawai S., Mori M., Uchiyama T., Isose S., Nasu S. (2010). Elevated CSF TDP-43 levels in amyotrophic lateral sclerosis: Specificity, sensitivity, and a possible prognostic value. Amyotroph. Lateral Scler..

[48] Kasai T., Tokuda T., Ishigami N., Sasayama H., Foulds P., Mitchell D.J., Mann D.M.A., Allsop D., Nakagawa M. (2009). Increased TDP-43 protein in cerebrospinal fluid of patients with amyotrophic lateral sclerosis. Acta Neuropathol..

[49] Steinacker P., Hendrich C., Sperfeld A.D., Jesse S., von Arnim C.A.F., Lehnert S., Pabst A., Uttner I., Tumani H., Lee V.M.-Y. (2008). TDP-43 in Cerebrospinal Fluid of Patients with Frontotemporal Lobar Degeneration and Amyotrophic Lateral Sclerosis. Arch. Neurol..

[50] Olsson B., Lautner R., Andreasson U., Öhrfelt A., Portelius E., Bjerke M., Hölttä M., Rosén C., Olsson C., Strobel G. (2016). CSF and blood biomarkers for the diagnosis of Alzheimer’s disease: A systematic review and meta-analysis. Lancet Neurol..

