# Supplementary material for: TDP-43 Cytoplasmic Translocation in the Skin Fibroblasts of ALS Patients
Source: Cells. 2022 Jan 8;11(2):209. doi: 10.3390/cells11020209 (PMC8773870; doi:10.3390/cells11020209)
Supplement: Supplementary file 1 [file cells-11-00209-s001.zip › cells-1515879-supplementary.pdf]

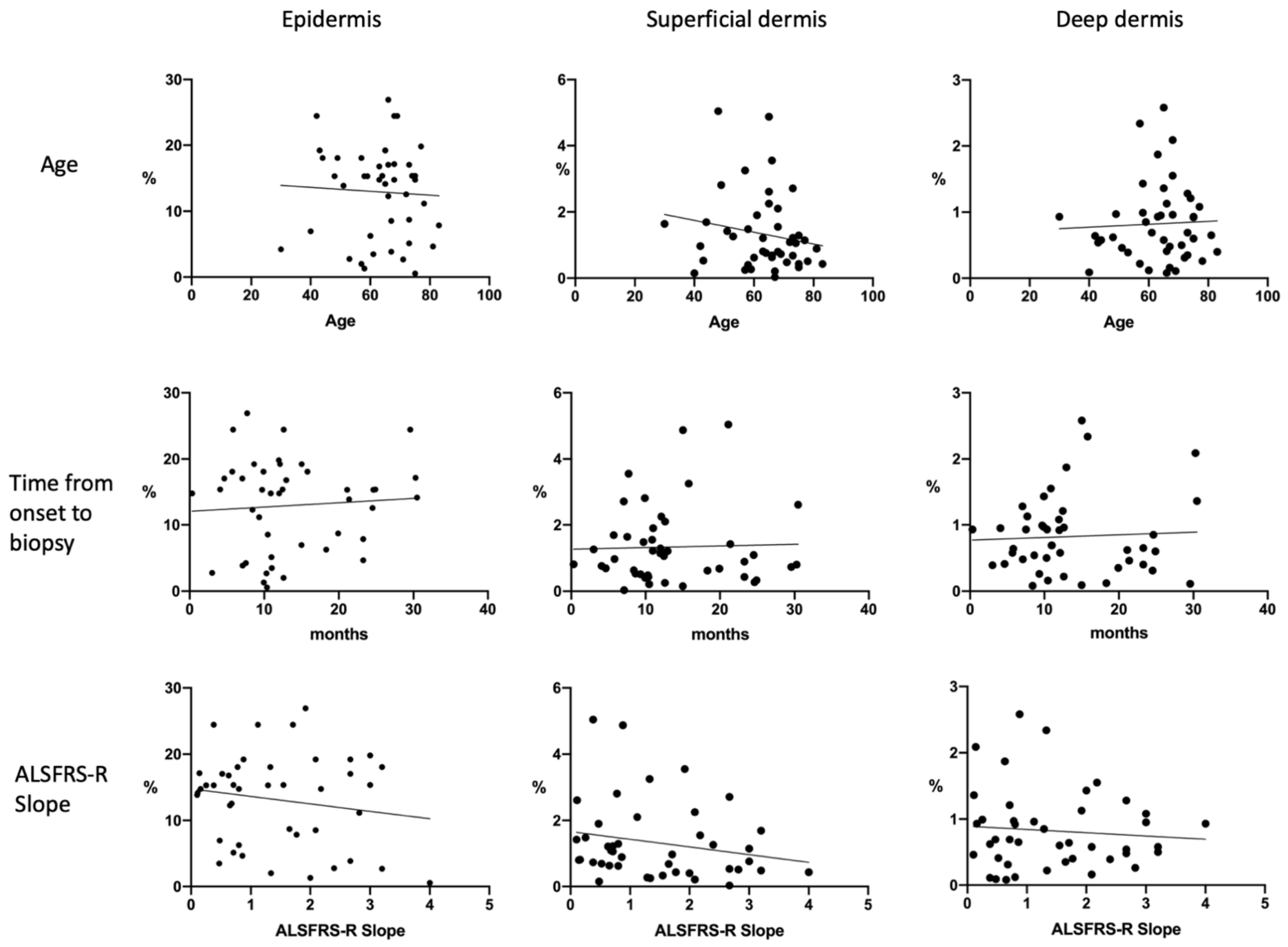

Figure S1. Correlation graphs of the percentage of cytoplasmic TDP-43 immunoreactivity with age, time from onset to biopsy and ALSFRS-R slope in the three different skin layers.
